# Triple‐Stimuli Responsive Soft Robots with Photo‐Programmable Ferriferous Oxide Particle Patterns

**DOI:** 10.1002/advs.202500669

**Published:** 2025-03-20

**Authors:** Siwei Hu, Kexing Li, Weijia Nong, Zhong‐Wen Liu, Zhao‐Tie Liu, Yanhu Zhan, Jinqiang Jiang, Peng Yang, Guo Li

**Affiliations:** ^1^ Key Laboratory of Syngas Conversion of Shaanxi Province Key Laboratory of Applied Surface and Colloid Chemistry Ministry of Education School of Chemistry and Chemical Engineering Shaanxi Normal University Xian Shaanxi 710062 China; ^2^ School of Materials Science and Engineering Liaocheng University Liaocheng 252000 China

**Keywords:** ferriferous oxide, soft robots, stimuli‐responsiveness

## Abstract

Magneto‐driven soft robots featuring remote and highly permeable controllability are considered promising, especially in biomedical and engineering applications. However, there is still lack of a high‐precision method to regulate the distribution of magnetic fillers in polymer substrates, which severely limits the improvement of the actuating functionality. This work provides a photo‐regulatable method to develop soft robots with locally distributed magnetic Fe_3_O_4_ nanoparticles. Solvent‐casted polyvinyl alcohol/sodium carboxymethyl cellulose film is prepared as the substrate, and Fe^3+^ ions are introduced to coordinate with carboxylate groups by surface treatment. Two processes, photo‐reduction of Fe^3+^ to Fe^2+^ ions and the hydrolytic reaction of the two ions, are sequentially combined to in situ generate magnetic Fe_3_O_4_ particles. Spatiotemporal control of UV light irradiation determines the Fe^3+^/Fe^2+^ ratio and, therefore the amount of generated Fe_3_O_4_ nanoparticles that decide magnetic field, NIR light, and moisture responsive actuating functionalities. Moreover, the external geometry of the composite can be tuned by inducing the formation of Al^3+^‐carboxylate coordinates for strain retention, which enables shape programming of the composite to exhibit complex 3D–3D actuating behaviors. The proposed method enables the design and preparation of soft robots with spatially tunable magnetism and more advanced actuating behaviors.

## Graphical abstract

The in situ generation of Fe_3_O_4_ nanoparticles in polymer substrate is realized by combining photo‐reduction of Fe^3+^ to Fe^2+^ and hydrolytic reaction of the two ions. By spatiotemporal control of UV light irradiation, the distribution of Fe_3_O_4_ nanoparticle can be well controlled, which enables the composite with magnetic field, NIR light, and moisture‐driven actuating behaviors.

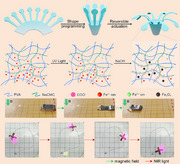

## 1 Introduction

Nature inspires the exploration of soft robots featuring their capability of controllable deformation and locomotion activated by external stimuli.^[^ ^1^, ^2^, ^3^, ^4^ ^]^ Of them, guiding the actuation of soft robots by magnetism is appealing because of the feasibility for remote and wireless control and high penetrability to human organs. These advantages render magnetic field‐responsive soft robots great potential in versatile applications, especially in medical and engineering scenarios.^[^ ^5^, ^6^, ^7^, ^8^ ^]^ Typical magneto‐driven soft robots comprise polymers as soft and elastic substrates and magnetic fillers (MFs) as functional ingredients. Commonly adopted MFs include ferrosoferric oxide (Fe_3_O_4_), neodymium‐iron‐boron (NdFeB), barium hexaferrite, cobalt, and chromium dioxide in the form of nano‐ or micro‐sized particles.^[^ ^9^, ^10^, ^11^, ^12^, ^13^, ^14^, ^15^, ^16^ ^]^ They endow the composites with actuating functionalities via generating attractive forces or inducing temperature fluctuations when exposed to external magnets or NIR light.^[^ ^10^, ^17^, ^18^, ^19^ ^]^ Therefore, the content and distribution of MFs in substrate are essential to the exhibited actuating functionalities. However, current design and preparation methods for regulating the distribution of MF in the substrate still lack enough precision. Considering the complex deformation existing in nature such as the synergic movement of octopus feet, developing methods to effectively regulate MF distribution is key to improve the actuating complexity of soft robots.

Typically, MF‐loaded soft robots are developed by either mixing MFs with polymer solutions followed by solvent evaporation, or by adding MFs into monomer solutions before initiating polymerization.^[^ ^12^, ^20^ ^]^ Methods for the surface functionalization of MFs are also applied to regulate their interactions with polymer chains.^[^ ^21^, ^22^ ^]^ However, these methods only result in homogeneous distribution of MFs in the composites. Recently, efforts are being made to regulate the alignment and distribution of MFs. For example, external magnets are applied to force the incorporated MFs to align along the direction of magnetic field, and the resulting composite exhibits direction‐differentiated physiochemical properties.^[^ ^23^, ^24^ ^]^ However, this method is only efficient in creating anisotropic features rather than regulating the distribution of MF. Moreover, the resolution of the resulting alignment patterns determined by the bulk size of the utilized magnets lacks enough precision.^[^ ^25^ ^]^ Ion transfer printing is also involved in spatially inducing the formation of MF particles in situ in polymer networks. However, the precision of the pattern determined by the utilized mold is still low.^[^ ^26^, ^27^ ^]^ In short, the creation of high‐resolution MF patterns in soft robots is still challenging.

**Figure 1:**
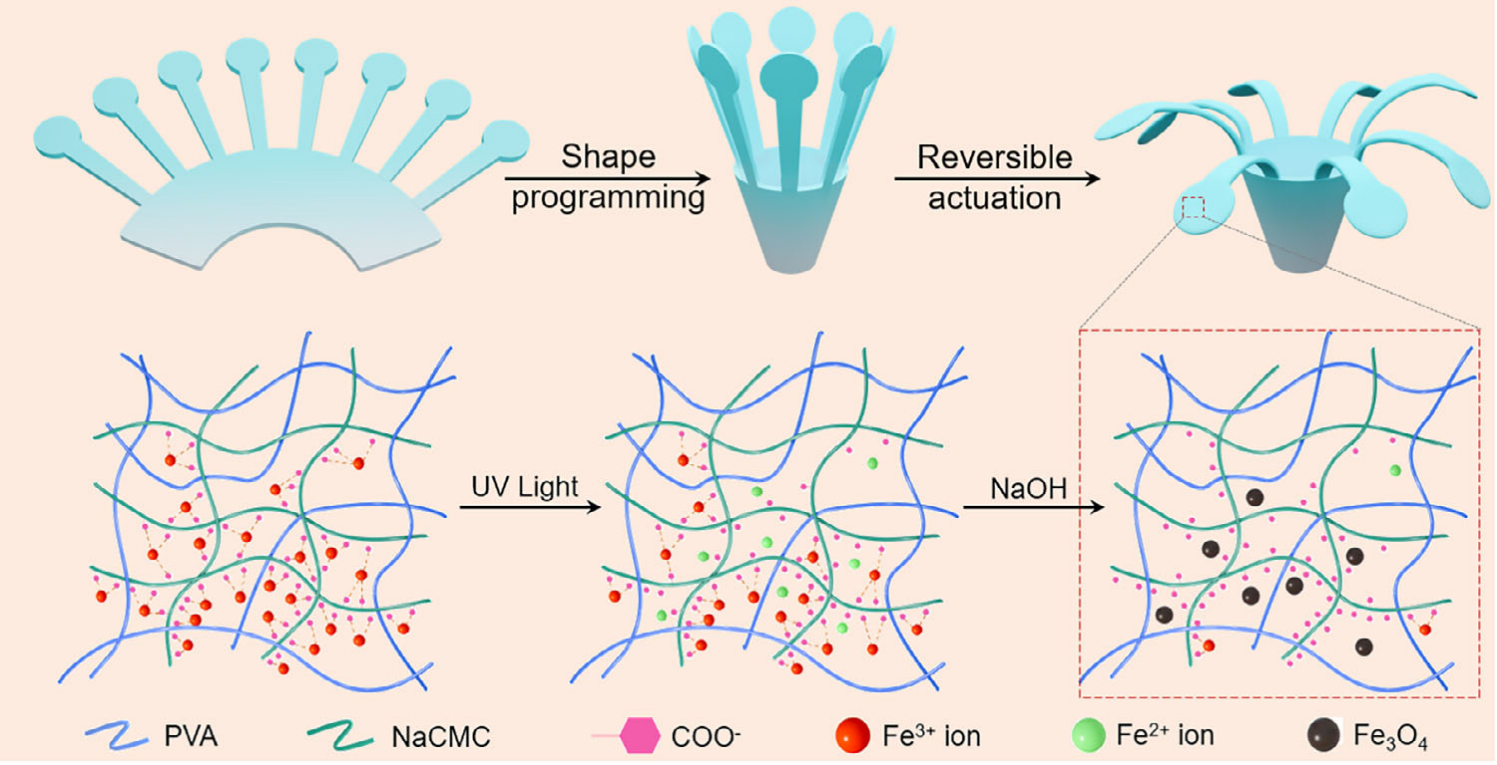
A scheme demonstrating the development of Fe_3_O_4_ nanoparticle concentration pattern that determines the actuation of the soft robot.

To address the challenge, we sought to utilize photo‐regulatable reactions to synthesize MFs in situ in polymer substrates. We noticed the fact that Fe^3+^ ions coordinated with carboxylate groups can be reduced to Fe^2+^ ions upon UV or visible light irradiation.^[^ ^28^, ^29^, ^30^, ^31^ ^]^ On another aspect, the hydrolytic reaction of Fe^3+^ and Fe^2+^ ions in alkaline conditions leads to the formation of ferroferric oxide (Fe_3_O_4_) particles.^[^ ^32^, ^33^ ^]^ Fe_3_O_4_ possesses superparamagnetic, good antioxidant, and excellent biocompatible properties and has been widely used as a typical material to endow soft robots with magnetic responsiveness.^[^ ^34^, ^35^, ^36^ ^]^ Therefore, a rational deduction is that if Fe^3+^‐carboxylate coordinates are introduced into polymer substrates, subjecting the material to UV light irradiation leads to partial photo‐reduction of Fe^3+^ ions, and further subjecting the irradiated material to base treatment can activate the hydrolytic reaction to generate Fe_3_O_4_. This strategy offers spatiotemporal light control to generate Fe_3_O_4_ patterns with enough precision. To demonstrate the availability of this idea, we prepare sodium carboxymethyl cellulose/polyvinyl alcohol blends by solvent casting. Polyvinyl alcohol (PVA) and sodium carboxymethyl cellulose (NaCMC) are used due to their good water solubility, excellent film‐forming property, and good biocompatibility, and the hydrogen bonds generated between them strengthen the formed blends. Fe^3+^ ions are introduced by a surface treatment step, and after successive UV light irradiation and alkaline treatment, Fe_3_O_4_ nanoparticles are generated in the surface‐treated region. The formation of Fe_3_O_4_ enables the developed composite magnetic field, NIR light, and moisture‐driven actuating functionalities. Moreover, by shape programming via inducing the formation of Al^3+^‐carboxylate coordinates as crosslinking for strain fixation, the actuating behaviors can be effectively regulated by altering external geometry, and different modes of actuating behaviors are realized. The process to develop soft robots with Fe_3_O_4_ concentration patterns is schematically presented in **Figure** **1**.

## 2 Results and Discussion

**Figure 2:**
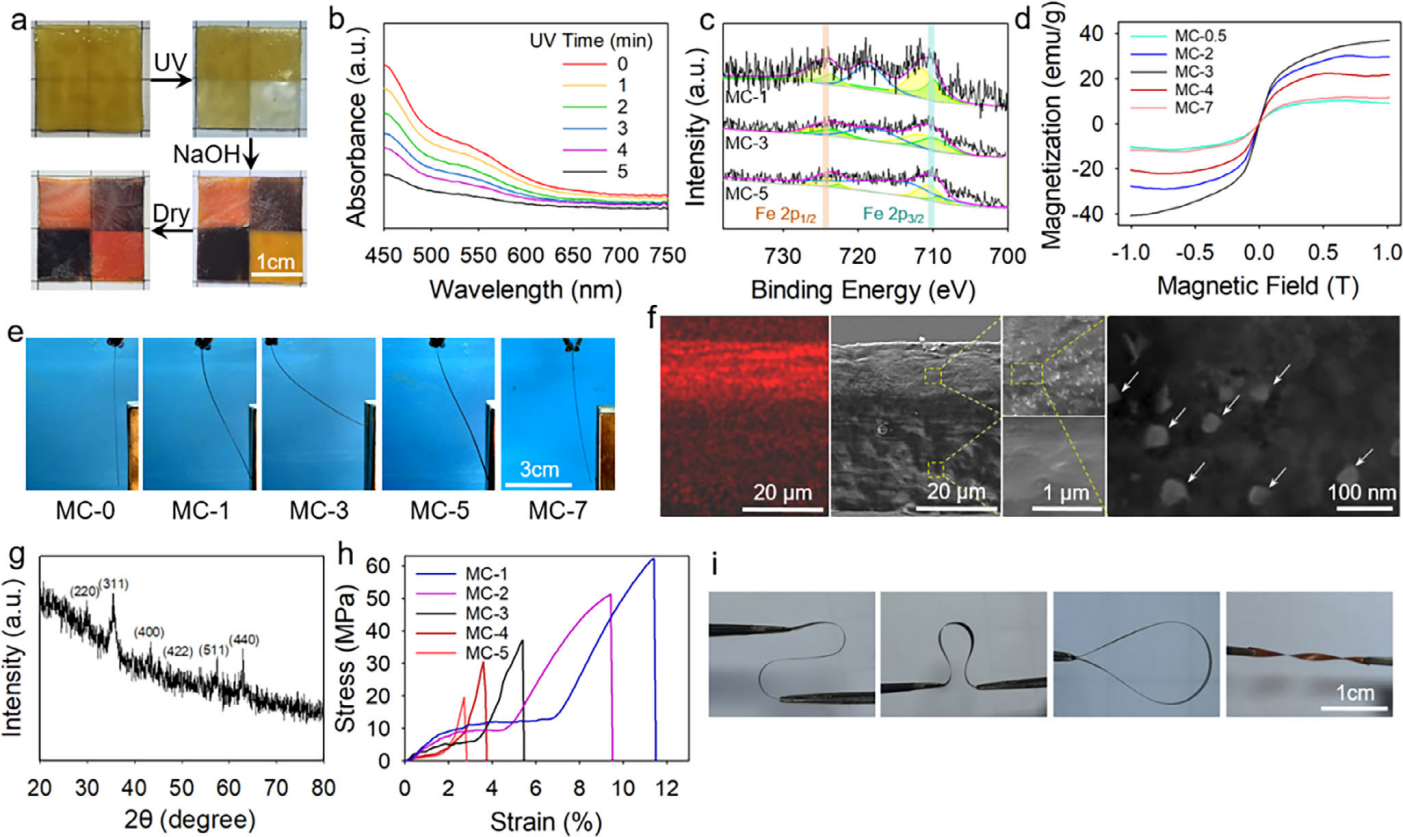
a) Images showing the process of developing a Fe_3_O_4_ pattern in a PVA/NaCMC sample. b) UV–vis spectra of surface‐treated PVA/NaCMC samples after different times of UV light irradiation. c) XPS‐spectra of MC‐1, MC‐3, and MC‐5. d) B–H hysteresis curves of samples with different UV light irradiation times. e) Images showing the maximum bending angle of magnetic field‐induced bending of samples with different UV light irradiation times. f) EDS mapping of Fe and SEM images with different magnifications showing the gradient distribution of Fe_3_O_4_ along the thickness direction of an MC‐3 sample. g) XRD curve of an MC‐3 sample. h) Stress–strain curves of samples with different UV light irradiation times. i) Images showing an MC‐3 strip that can tolerate different types of deformation.

The images presented in **Figure** **2**a show the developing process of a Fe_3_O_4_ pattern in situ in the PVA/NaCMC blending substrate. The substrate is prepared by dissolving the two polymers in deionized water, followed by a freezing/thawing process to induce the crystallization of PVA chains as physical crosslinking. The physical hydrogel is surface treated using an aqueous solution of ferric chloride (Fe^3+^ solution) to introduce Fe^3+^ ions into the substrate for the formation of Fe^3+^‐carboxylate tri‐coordinates with NaCMC chains. Further subjecting to UV light irradiation by varying the irradiation time and position, the extent of photo‐reduction of Fe^3+^ to Fe^2+^ is spatially differentiated. The irradiated hydrogel sample is then immersed into a 8% NaOH aqueous solution, allowing the happening of hydrolytic reaction to generate Fe_3_O_4_. The spatial difference in the Fe^3+^/Fe^2+^ ratio leads to a concentration pattern of formed Fe_3_O_4_ particles in the substrate. Finally, the hydrogel is dried to obtain the target composite. In the following context, we use MC‐*x* to indicate samples or regions with Fe_3_O_4_ patterns, where *x* is the irradiation time (min). The formation of the Fe_3_O_4_ pattern can be visually detected by the naked eyes because of its difference in color, as regions with high Fe_3_O_4_ concentrations exhibit darker colors, whereas low Fe_3_O_4_ concentration regions are in redder colors. The effectiveness of photo‐reduction of Fe^3+^ to Fe^2+^ ion is verified by UV–vis spectroscopy, as the intensity of the curves of samples with prolonged irradiation times gradually decrease (Figure 2b). Figure 2c shows the XPS spectra of MC‐1, MC‐3, and MC‐5. Two peaks corresponding to Fe 2p_3/2_ and Fe 2p_1/2_ can be observed at binding energies of 711.0 and 724.5 eV in all samples, which are the characteristic peaks of Fe^3+^ and Fe^2+^ ions, respectively. For the fitted peaks, the ones at binding energies of 710.5 and 724.5 eV are assigned to Fe^2+^, and that of 712.0 and 725.0 eV belong to Fe^3+^.^[^ ^37^, ^38^ ^]^ Moreover, the difference of the samples lies in that the Fe^2+^/Fe^3+^ ratio determined by integrating the area of corresponding peaks is in the order of MC‐3>MC‐1>MC‐5, showing a time‐dependent tendency that first increases and further decreases. This can be explained as that Fe^2+^ ions only exist in Fe_3_O_4_ in the developed composite, as Fe^2+^ ions would be oxidized to +3 valence if they do not participate in the hydrolytic reaction to form Fe_3_O_4_.^[^ ^39^, ^40^ ^]^ Since there exists an optimistic irradiation time to have the optimum Fe^2+^/Fe^3+^ ratio to form the ideally largest amount of Fe_3_O_4_, less or excessive irradiation leads to excessive Fe^3+^ or Fe^2+^ ions that do not participate in the hydrolytic reaction. In the case of prolonged irradiation, the unreacted Fe^2+^ ions can be further oxidized into Fe^3+^ ions in an alkaline environment, leading to a lower value of detected Fe^2+^/Fe^3+^ ratio. Therefore, the value of the Fe^2+^/Fe^3+^ ratio determines the magnetism of the resulting composite. This is evidenced by surveying the B–H hysteresis curves of samples with different UV light irradiation times (Figure 2d), which shows an increase‐then‐decrease tendency, and MC‐3 shows the strongest magnetism. The difference in magnetism is also verified by approaching the samples to a magnet and observing the largest bending angle. As the results presented in Figure 2e, the value of bending angle increases and then decreases for samples with increasing irradiation time, and MC‐3 shows the most prominent bending deformation. These results indicate that a proper UV light irradiation time is critical in obtaining a composite with strong magnetism, and in our investigated situation 3 min is the optimum irradiation time. Besides controlling the distribution in planer directions, Fe_3_O_4_ nanoparticles also distribute in a gradient manner along the thickness direction. Figure 2f shows the SEM and corresponding EDS mapping of Fe along the thickness direction of an MC‐3 sample. The uneven distribution of the red signal indicates that Fe^3+^ ions mainly locate at the regions near the treated surface. Moreover, the highly bright Fe_3_O_4_ nanoparticles, with diameters lower than 100 nm, can be clearly observed in the Fe‐rich region in the SEM image, whereas the inner region with a weak Fe signal intensity exhibits a smooth morphology without any observable Fe_3_O_4_ nanoparticle. The crystalline structure of the generated Fe_3_O_4_ nanoparticles is assured by X‐ray diffraction spectroscopy. The characteristic peaks in the XRD spectrum of MC‐3 presented in Figure 2g locate at 2*θ* = 29.9°, 35.5°, 43.2°,53.5°, 56.9°, and 62.3°, which are assigned to (220), (311), (400), (422), (511), and (440) crystal faces of Fe_3_O_4_ crystals, respectively.^[^ ^41^ ^]^ We further investigate the mechanical properties of the composite after the generation of Fe_3_O_4_ nanoparticles. The stress–strain curves presenting in Figure 2h suggest that a longer time of UV light irradiation deteriorates the network toughness, as with increasing the irradiation time from 0 to 5 min, the fracture strain decreases from 11.50% to 2.85%, accompanied by the simultaneous decrement of tensile strength from 62.22 to 19.55 MPa. This tendency is possibly due to that the photo‐reduction of Fe^3+^ to Fe^2+^ ions induces decrosslinking of the network, which not only deteriorates network strength but also impairs effective stress transmission and dissipation. Moreover, the formed Fe^2+^ ions may enhance hygroscopicity of the network, and the increase in equilibrium water content also leads to decrease in mechanical properties. It should be noted that although the extensibility after Fe_3_O_4_ pattern formation is compromised, the material can tolerate other types of deformation, such as bending, twisting, and torsional deformation, without fracture (Figure 2i). Unless otherwise stated, MC‐3 is used for the following investigation.

**Figure 3:**
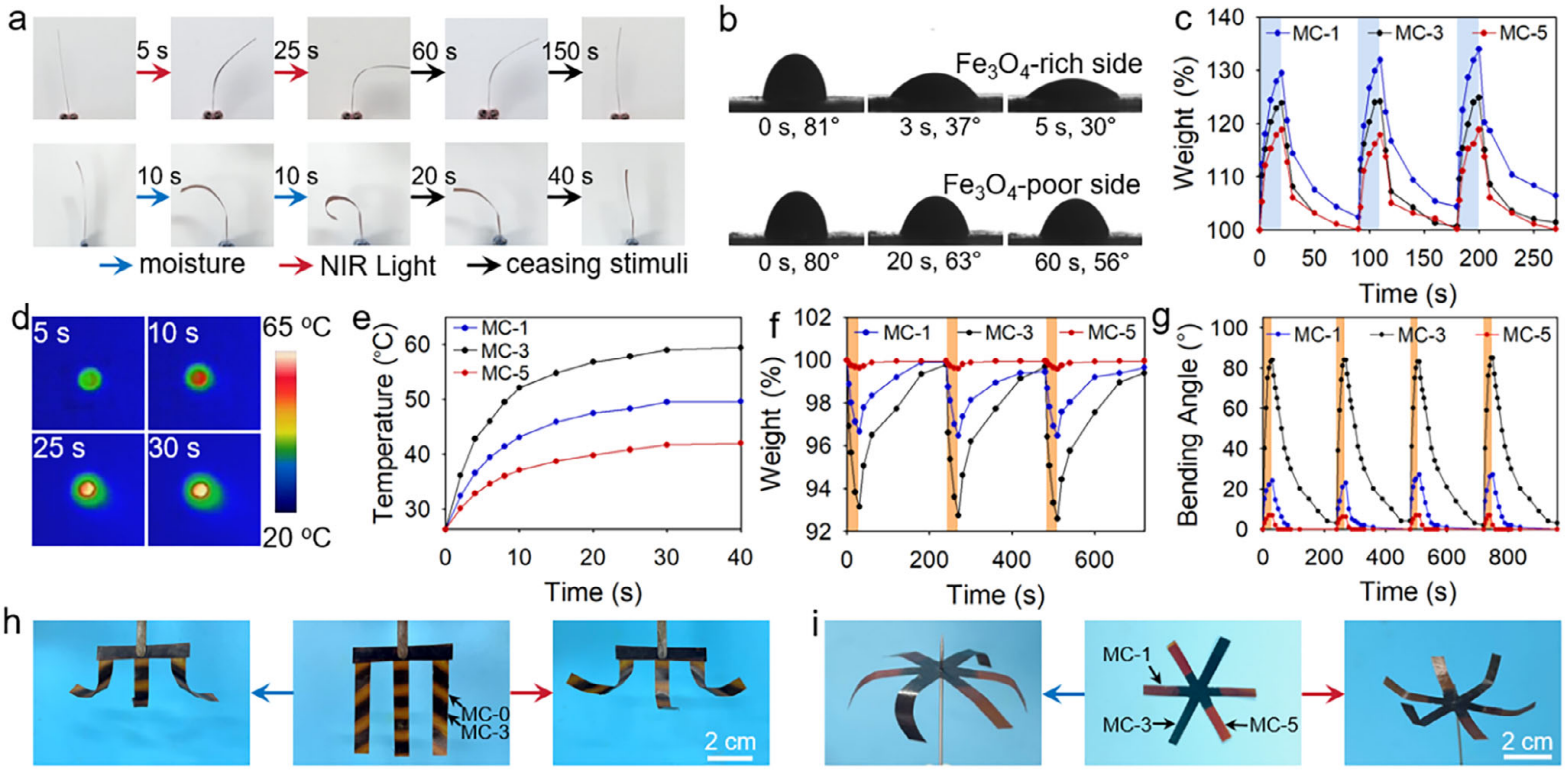
a) Images showing the bidirectional bending deformation of an MC‐3 strip triggered by NIR light and moisture. b) Time‐dependent changes of water contact angles of the two sides of an MC‐3 sample. c) Weight fluctuations of MC‐1, MC‐3, and MC‐5 upon cyclic moisture stimulation. d) Infrared thermal images showing the temperature rise of an MC‐3 sample under NIR light irradiation. e) plots showing the temperature rise of MC‐1, MC‐3, and MC‐5 under NIR light irradiation. f) Weight fluctuations and g) corresponding changes of bending angle of MC‐1, MC‐3, and MC‐5 under cyclic NIR light exposure. h,i) Images showing designated actuating behaviors of samples with Fe_3_O_4_ patterns triggered by NIR light and moisture.

Besides tunable magnetism, the in situ generation of Fe_3_O_4_ particles endows the composite NIR light and moisture‐responsive actuating behaviors. The photos in **Figure** **3**a exhibit the actuating behaviors of an MC‐3 strip under NIR light and moisture stimulation, respectively. When exposed to NIR light, the strip quickly deforms from its initial straight shape to a 83° bending angle toward the Fe_3_O_4_‐rich side, which further recovers to its initial straight shape in 150 s after ceasing irradiation. It should be noted that the direction of the incident light does not affect the actuating behavior, as the resulting bending angle by irradiating from the Fe_3_O_4_‐rich side and the Fe_3_O_4_‐poor side are quite approaching (83° and 79°). When humidified, the sample exhibits a bending deformation toward the Fe_3_O_4_‐poor side, and after reaching a maximum bending angle of 54°, the sample can restore its initial straight shape in 60 s upon drying. This interesting bidirectional bending deformation originates from the reversible water absorption/desorption of the material with changing the surrounding environment. To verify the actuating mechanism, the effect of Fe_3_O_4_ generation on the hygroscopicity of the composite is surveyed. Images presented in Figure 3b show the water contact angle (WCA) of the Fe_3_O_4_‐rich and Fe_3_O_4_‐poor sides. The initial value of WCA on the Fe_3_O_4_‐rich side is 81°, which rapidly decrease to 30° after 5 s of stay of the water droplet. For the Fe_3_O_4_‐poor side, although the value of WCA after immediate contact is 80°, quite approaching to that of the Fe_3_O_4_‐rich side, it decreases to 56° after 60 s of stay of the water droplet, exhibiting a much less prominent extent of decrease of WCA. This results suggest that the Fe_3_O_4_‐rich region is more hygroscopic and can absorb moisture much faster than the Fe_3_O_4_‐poor region. Weight fluctuations of samples with varying Fe_3_O_4_ contents upon cyclic RH change are also surveyed. The results given in Figure 3c show that samples with prolonged UV light irradiation times exhibit less prominent amplitude of weight change. The enhancement in hygroscopicity after Fe_3_O_4_ particle generation is due to the consumption of highly hydrophilic carboxylate groups and the accumulation of hygroscopic NaCl salt in the Fe_3_O_4_‐rich side. The consumption of carboxylate groups is verified by FT‐IR, as the intensity of the characteristic peak assigning to symmetric stretching vibration of carboxylate groups decreases after irradiation (Figure S1, Supporting Information), and the evidence of preferred accumulation of NaCl is confirmed by EDS mapping of Na along the thickness direction of a MC‐3 sample (Figure S2, Supporting Information). This leads to a prominent change in equilibrium water content of the film in different RH environments, enabling the composite to exhibit prominent moisture‐driven actuating behavior (Figure S3, Supporting Information). The effect of thickness on the actuating behavior is investigated, and the film thickness is adjusted for guatenting prominent actuating behaviors (Figure S4, Supporting Information). The photothermal behaviors of the composite are next surveyed. Figure 3d presents the infrared thermal images of an MC‐3 sample upon NIR light irradiation. The central temperature of the irradiated region quickly rises from 26.3 to 58.9 °C within 30 s, and a notable temperature gradient from center to edge can be clearly observed. The amplitude of temperature rise is closely related to the Fe_3_O_4_ concentration, as after 30 s of irradiation the maximum temperature of MC‐3 is 60.4 °C, whereas that of MC‐1 and MC‐5 is only 49.5 and 41.9 °C, respectively (Figure 3e). This suggests that the NIR light‐driven actuating behavior can be effectively regulated by controlling the extent of temperature rise via a Fe_3_O_4_ concentration pattern. Besides, the actuating behavior can also be regulated by controlling the NIR light intensity (Figure S5, Supporting Information). The mechanism of NIR light‐driven actuation can be explained as follows NIR light exposure induces more prominent temperature change in the Fe_3_O_4_‐rich regions, leading to larger extents of water loss/capture and corresponding volume change. Therefore, the gradient distribution of Fe_3_O_4_ leads to bending deformation toward the Fe_3_O_4_‐rich side under irradiation. This mechanism is verified by testing NIR light‐induced weight change and corresponding bending behaviors of samples with different UV light irradiation times. As the results given in Figure 3f,g, MC‐3, the one with the highest amount of Fe_3_O_4_, exhibits the most prominent weight fluctuation (7.17%) and actuating behavior compared with other samples under cyclic NIR light exposure. These results also suggest that by controlling the concentration of Fe_3_O_4,_ the amplitude of actuation can be well regulated. Based on these results, we next show that the direction and amplitude of actuation can be spatially regulated by the developed Fe_3_O_4_ pattern. Two examples are presented in Figure 3h and 3i for demonstration. The first sample is first processed into a letter “E” like shape, and Fe_3_O_4_ patterns with different tilting angles are developed on each of the three arm. Upon actuation by moisture and NIR light, the three arms reversibly bend toward different directions. For the second example, the 6 arms are divided into 3 pairs and for each pair Fe_3_O_4_ with a certain concentration is developed. Upon the stimulation of moisture and NIR light, the difference in Fe_3_O_4_ concentration leads to different bending angles of the three pairs of arms.

**Figure 4:**
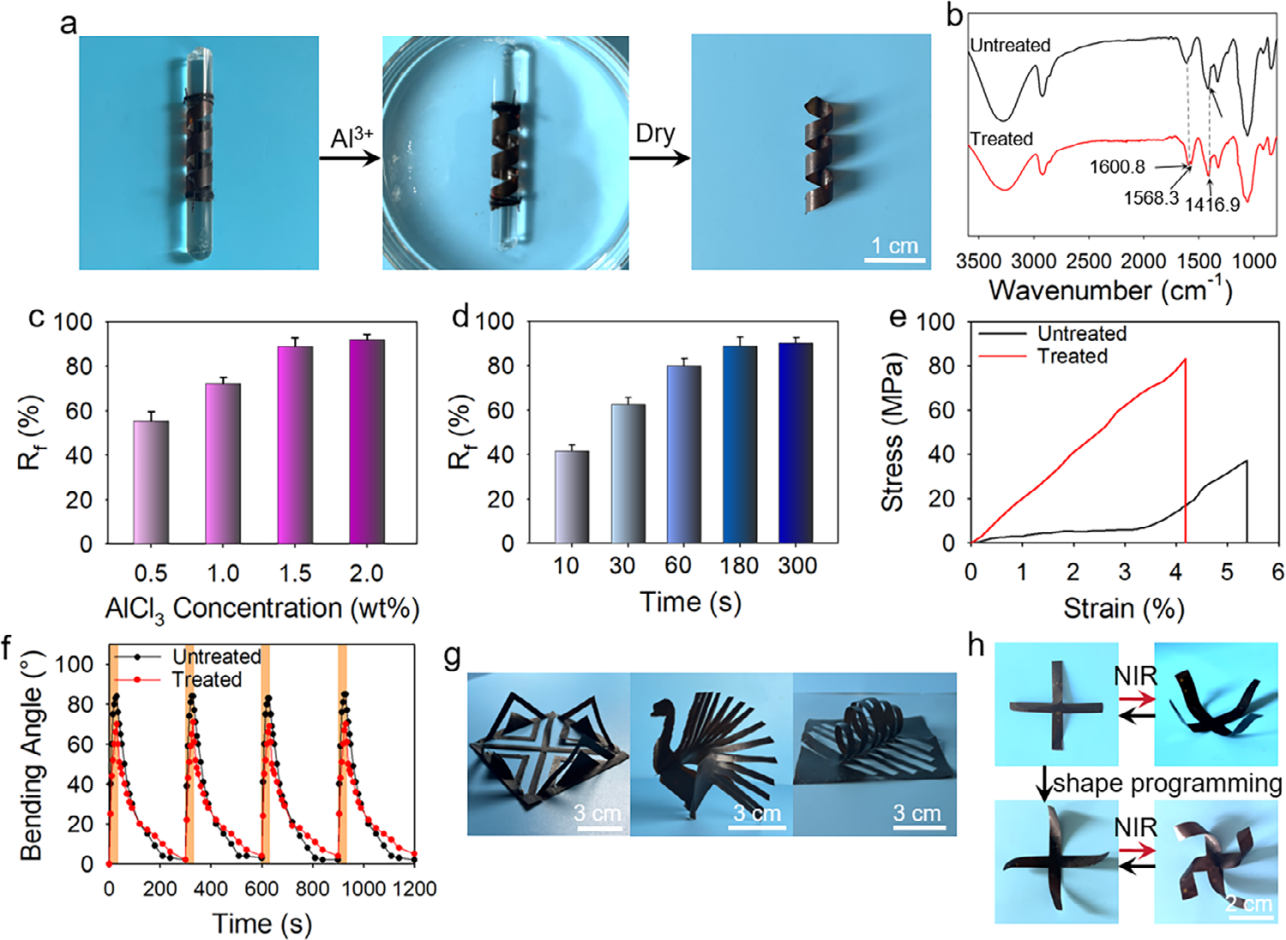
a) Images showing shape programming process of an MC‐3 strip. b) FT‐IR spectra of an MC‐3 sample before and after treatment. The effects of c) Al^3+^ concentration and d) treatment time on R_f_. e) Stress–strain curves, and f) NIR light‐induced actuation of MC‐3 before and after treatment. g) Images showing MC‐3 samples with programmed 3D shapes. h) Images showing NIR light‐induced actuating behaviors of a MC‐3 sample before and after shape programming.

On another front, the external geometry of the composite can be programmed by inducing the formation of multivalent metal‐carboxylate coordinates as crosslinking for strain fixation. A typical process can be found in **Figure** **4**a, as a strip of MC‐3 is first deformed into a twisted shape on a glass rod, and the sample and the glass rod are together immersed in an aqueous solution containing aluminum chloride. After 3 min, the sample is taken out from the solution and separated from the rod, and a stable twisted shape is obtained. Mechanistically, this immersion step allows the diffusion of Al^3+^ ions into the composite and coordinate with carboxylate groups, and the formed coordinates act as crosslinking points to retain the elastic recovery of the deformation. The formation of coordinating structure is verified by FT‐IR spectroscopy, the characteristic peaks at 1620.7 and 1421.1 cm^−1^ in the spectrum of the untreated sample are assigned to the symmetric and asymmetric stretching vibration of the ‐COO^−^ group, respectively. The respective blue shift of the two peaks to 1600.8 and 1416.9 cm^−1^ in the spectrum of the treated sample proves the coordination of carboxylate groups with Al^3+^ ions.^[^ ^42^, ^43^, ^44^ ^]^ The strain fixation efficiency is quantified by strain fixation ratio (R_f_), which is determined as the percentage of fixed strain comparing with the total strain. The effects of relevant parameters is next investigated to optimize the strain fixation process and to obtain the highest R_f_. Figure 4c,d show R_f_s obtained from MC‐3 samples after immersing into aluminum chloride solutions with different concentrations for 300 s or immersing in a 1.5 m aluminum chloride solution for different times. The results clearly show that higher Al^3+^ concentration or longer immersing time leads to better strain fixation, and the highest R_f_ can be 91.3% or 89.1% in each situation. In the following investigations, the concentration of Al^3+^ solution is fixed at 1.5 m and a 180 s of immersion process is utilized. The formation of Al^3+^‐carboxylate coordinates improves the mechanical properties, as in tensile tests, the tensile strain after coordination increases from 37.7 to 83.1 MPa, while the tensile strain only moderately decreased from 5.39% to 4.18%. This indicates that the formed coordinates facilitate transmission and dissipation of internal stress in the network to avoid crack formation.^[^ ^45^ ^]^ We also show that the formation of coordination structures has very limited influence on the actuating behavior, as under cyclic NIR light exposure, the amplitude of bending actuation mildly decreases from 83° to 70° (Figure 4f). We further demonstrate that by this one‐step immersion process, composite with originally processed 2D shapes can be programmed into delicate 3D shapes, as the three examples presented in Figure 4g. The 3D shape programming of the composite enables regulation of the actuating behavior. An example for demonstration can be found in Figure 4h, after programming the originally straight 4 arms of a cross‐like MC‐3 sample into curled geometries, the actuating behavior changes correspondingly from bending into curling actuation.

**Figure 5:**
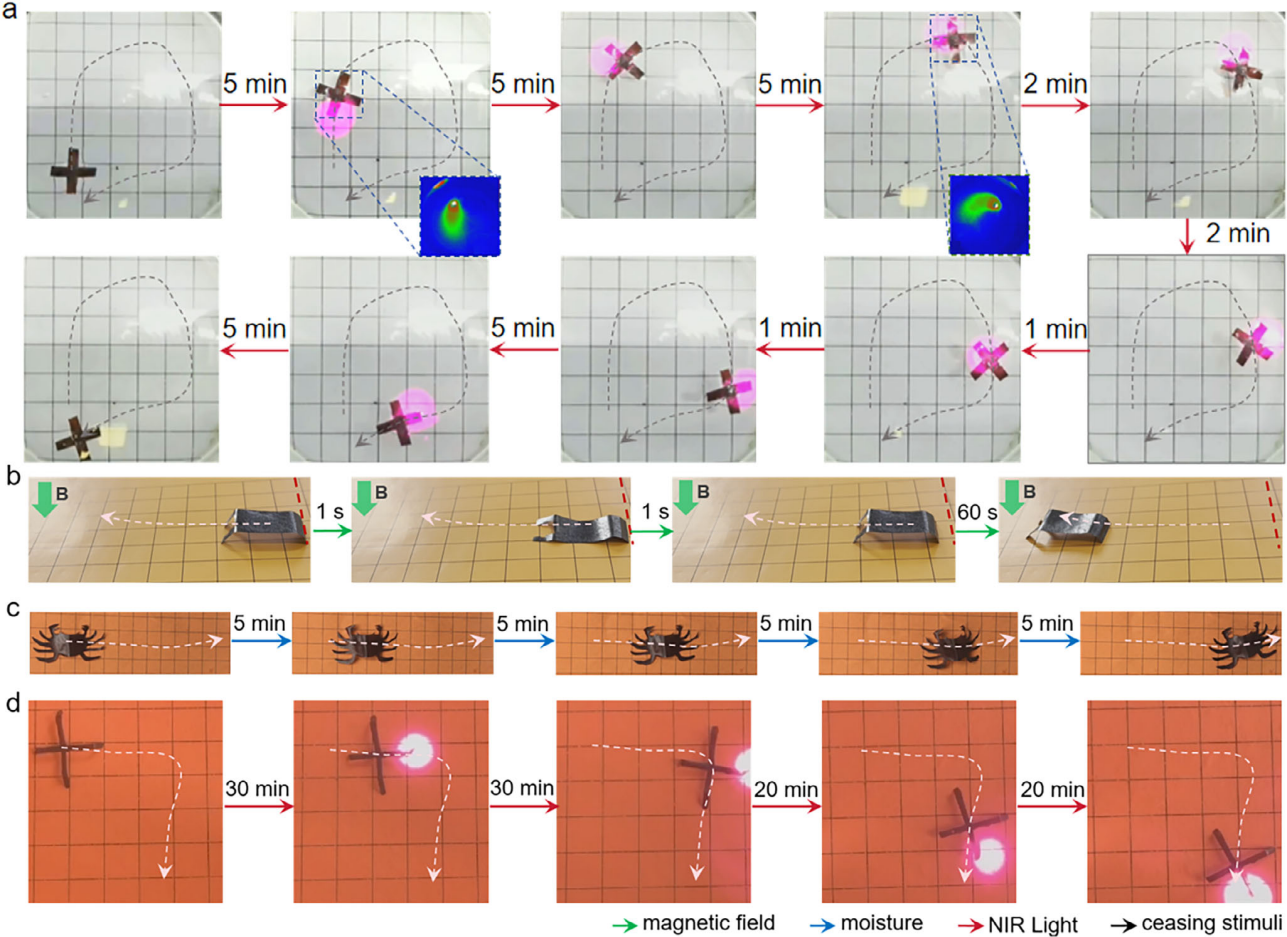
a–d) 4 MC‐3 samples with programmed 3D shapes exhibiting different modes of actuating behaviors activated by magnetic field, moisture, and NIR light. The moving trajectories of the samples are denoted using dotted lines.

The multi‐stimuli driven actuation and shape programming functionalities are combined to realize more flexible actuating behaviors. 4 examples are presented in **Figure** **5**. In the first case, we show that an MC‐3 sample with a cross‐shape floating on water can be driven by NIR light to travel at the water‐air interface. By selecting the position to irradiate, the sample moves in a circle around the internal edge of the round container and finally go back to the starting position. The actuating mechanism is based on the Marangoni effect, which relies on the temperature rise of the sample and heat transfer from the sample to the surrounding environment.^[^ ^46^, ^47^, ^48^ ^]^ The heat transfer forms a temperature gradient that changes the surface tension of water, resulting in water flow that drives the sample to move. Second, we show that a four‐foot actuator with programmed asymmetrical geometry can be driven to walk by the magnetic field. By asymmetric shap programming, the two front legs exhibit bending deformation more prominent than the two hind legs, leading to a larger amplitude of reversible bending actuation of the front legs when alternatively approaching and deviating from an external magnet. As a result, the sample continuously moves forward. Similarly, exposing a crab‐like sample with asymmetrically programmed legs to intermittent spraying of moisture, the sample moves horizontally along the pre‐designed direction. We also show that a cross‐shaped MC‐3 sample, with its for legs programming into bending geometries and being spatially irradiated by NIR light to trigger the actuation of a certain leg, the sample controllably moves in planar directions on the flat surface. The movement of samples presented in Figure 5a,c can also be found in videos in Supporting Information.

**Figure 6:**
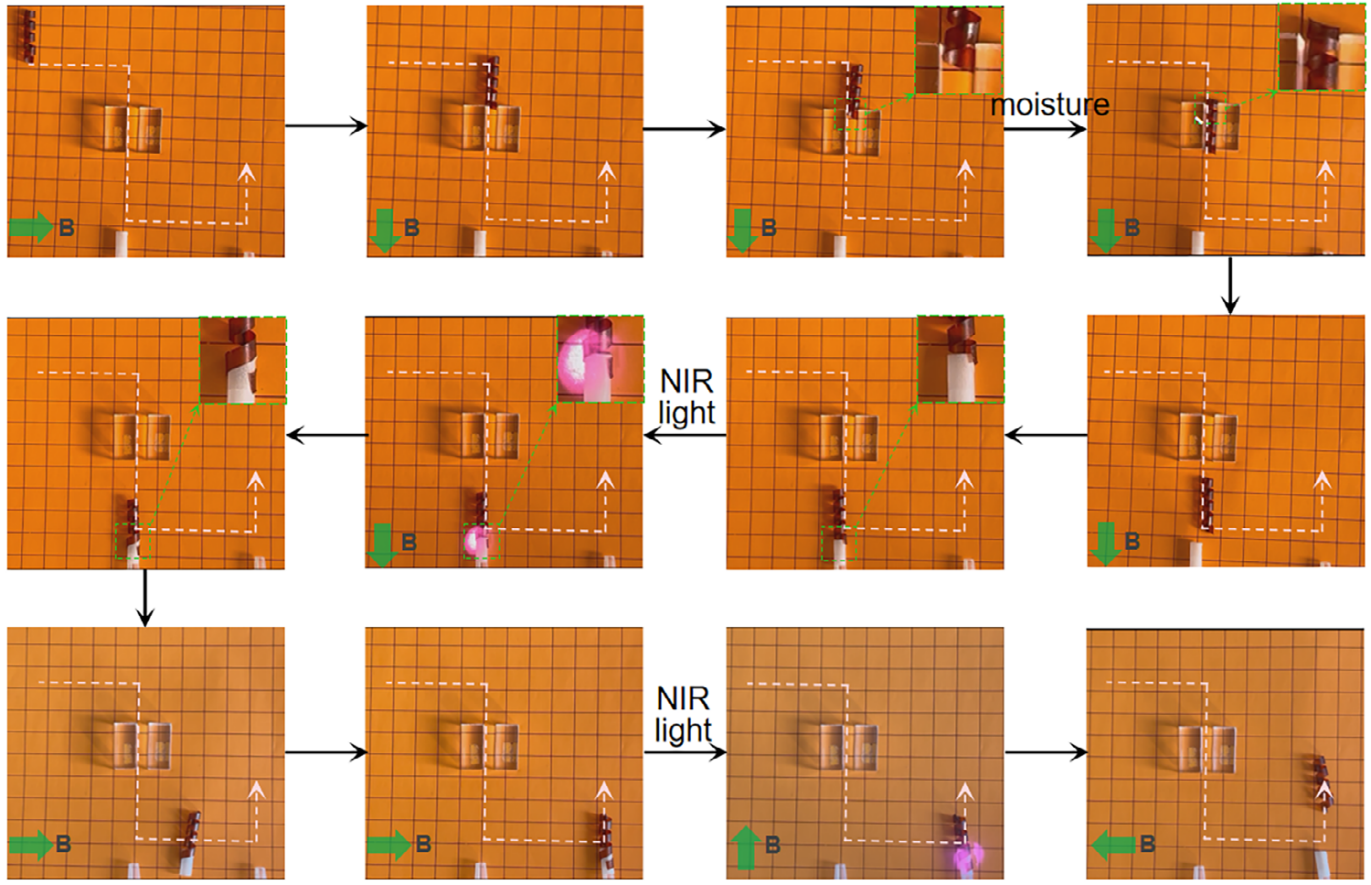
The execution of complex tasks of an MC‐3 spiral under synergic control of moisture, NIR light, and magnet.

We further show that by synergistically utilizing the three stimuli, the sample can be controlled to execute complex tasks. As images presented in **Figure** **6**, the sample with a spiral shape can be attracted by an external magnet to freely travel on the flat surface until stuck by a slit. To successfully pass through, the sample is exposed to moisture. Since the outer surface is Fe_3_O_4_‐rich, asymmetric water absorption drives the sample to further spiral, which is accompanied by the decrease of its diameter to a value lower than that of the slit. This allows the successful pass of the sample through the slight driven by magnet, and ceasing moistening the sample recovers to its original diameter. After approaching a silicon rubber tube inserted on a rod, NIR light is applied to induce the expansion of the spiral, and the temporal increase in diameter allows the sample to clamp the tube. Further applying the magnet to drive the sample to rotate to another rod, the tube is released by exposing the sample to NIR light irradiation again to trigger an reversible expansion deformation, and then the spiral moves to the final position, and the task is completed. This example clearly demonstrate that the developed actuator is capable to adapt to different environmental conditions and execute complex tasks via spatiotemporal control of the external geometry and the actuating behavior upon proper stimulation. A video showing the process shown in Figure 6 can be found in the Supporting Information.

## 3 Conclusion

This work demonstrates an effective way to develop magneto‐driven soft robots with spatially tunable magnetism. Fe^3+^ ions are introduced to a thin film of PVA/NaCMC blend by surface treatment, resulting in the gradient distribution of Fe^3+^ ions. Fe_3_O_4_ particles with spatially differentiated concentrations are successfully developed in situ in the thin film by partial photo‐reduction of Fe^3+^ to Fe^2+^ ion and hydrolytic reaction of the the two ions. The results show that the amount of generated Fe_3_O_4_ particles is closely related to the time of UV light irradiation, which further affects magnetic field, NIR light, and moisture responsive behaviors of the composite. By spatiotemporal control of UV light irradiation, concentration patterns of Fe_3_O_4_ particles are successfully created in the composite, which enables designable multi‐stimuli responsive actuating behaviors. Moreover, the geometry of the composite can be programmed by inducing the formation of Al^3+^‐carboxylate coordinate as physical crosslinking, allowing the fixation of deformation. By doing this, composites with planer shapes can be deformed into intricate 3D shapes, and by orthogonal designing of geometry and Fe_3_O_4_ pattern, multi‐mode reversible deformation and locomotion triggered by external magnet, NIR light, and moisture are realized. This work provides a high‐precision strategy to develop soft robots with spatially tunable magnetism for advanced actuating functionalities and extended application scenarios.

## 4 Experimental Section

### Materials

Iron chloride hexahydrate (FeCl_3_·6H_2_O) was purchased from Shanghai Aladdin Biochemical Technology Co., Ltd. Polyvinyl alcohol (1799) was purchased from Chengdu Aike Reagent Co., Ltd. Sodium carboxymethyl cellulose (NaCMC, 600–3000 MPa (2%) at 20 °C) was purchased from Shanghai Macklin Biochemical Technology Co., Ltd. Anhydrous ethanol (CH_3_CH_2_OH) and Aluminum trichloride hexahydrate (AlCl_3_·6H_2_O) were purchased from Guangdong Guanghua Sci‐Tech Co., Ltd. Sodium hydroxide (NaOH) was purchased from China National Pharmaceutical Group Chemical Reagent Co., Ltd.

### Preparation of PVA/NaCMC Blend

PVA and NaCMC in the weight ratio of 2:1 were dissolved in deionized water by continuous stir. Afterward, the solution was filled into a homemade silica mold (90 × 90 × 1 mm) and fixed using clips. After that, the mold was freezed at −20 °C for 12 h, followed by thawing at room temperature to obtain the blend.

### Development of Fe_3_O_4_ Pattern

The prepared blend was surface treated using filter papers soaked with ferric chloride aqueous solution (0.15 m) for 12 min. After that, the filter paper was removed, and the excessive liquid on the sample surface was gently removed by wiping. UV light irradiation (374 mW cm^−2^) was then applied for a certain time, and the irradiated hydrogel was immersed in a NaOH solution (1.5 m) at 50 °C for 30 min. The hydrogel was then taken out and washed using a 30 wt.% ethanol solution to a neutral pH, followed by drying at 45 °C for 4 h to obtain the target materials.

### The Measurement of R_f_

Typically, samples were first processed into strips with the same geometries (50 mm × 5 mm × 0.05 mm). They are deformed into a 90° bending angle and subsequently inserted into a mold, which were together immersed into aluminum chloride aqueous solution. The concentration of the solution and the immersion time could vary to optimize the fixation efficiency. Subsequently, the samples were gently wished using deionized water and dried in a 60 °C oven for 5 min to obtain samples with fixed bending deformation (θ). *R_f_* was calculated as the percentage ratio of θ to 90°.

### Characterizations

The crystalline structure of the composites was characterized by X‐ray diffraction (XRD, SmartLab 9, Japan). Fourier transform infrared spectra (FTIR) were obtained using a Thermo Scientific FTIR spectrometer (PerkingElmer Frontier FTIR) in the range of 4000–400 cm^−1^ and an accumulation of 16 scans per analysis. X‐ray photoelectron spectroscopy (XPS) measurements were conducted on an AXIS ULTRA multifunctional spectrometer (Kratos Analytical Ltd, Kratos, U.K.), and the binding energy of C 1s was shifted to 284.8 eV as the reference. The microstructure of the composites was investigated by using field emission scanning electron microscopy (SEM) (SU8220, Hitachi, Japan. Operating voltage: 20 kV). The tensile behaviors of the films were investigated by using a Suns UTM2103 (Shenzhen Suns, China) universal tensile test machine with a 100 N load cell. The water contact angles were measured using a contact angle measuring system (OCA 20, Dataphysics, Germany), and the volume of the water drop was 2 µL controlled by pipettes. Diffuse reflectance spectroscopy spectra were recorded on a UV–vis–NIR spectrometer (UV3600, Shimadzu, Japan) by placing samples on a transparent quartz flake and taking scans from 200 to 800 nm. The magnetization evaluation was carried out with vibrating sample magnetometer (VSM) at room temperature (Iran Meghnatis Daghigh Kavir Company). An infrared thermal imaging camera (HM‐TPH21Pro‐3AQF, HIKMICRO, operating voltage: 5 kV) was used to record the temperature changes upon NIR light irradiation.

## Conflict of Interest

The authors declare no conflict of interest.

## Supporting information



Supporting Information

## Data Availability

The data that support the findings of this study are available from the corresponding author upon reasonable request.
