# Supplementary material for: Triple‐Stimuli Responsive Soft Robots with Photo‐Programmable Ferriferous Oxide Particle Patterns
Source: Adv Sci (Weinh). 2025 Mar 20;12(18):2500669. doi: 10.1002/advs.202500669 (PMC12079459; doi:10.1002/advs.202500669)
Supplement: Supplementary file 1 — Supporting Information [file ADVS-12-2500669-s001.docx]

Supporting Information

Triple-stimuli responsive Soft Robots with Photo-Programmable Ferriferous Oxide Particle Patterns

Siwei Hu, Kexing Li, Weijia Nong, Zhong-Wen Liu, Zhao-Tie Liu, Yanhu Zhan, Jinqiang Jiang, Guo Li^*^


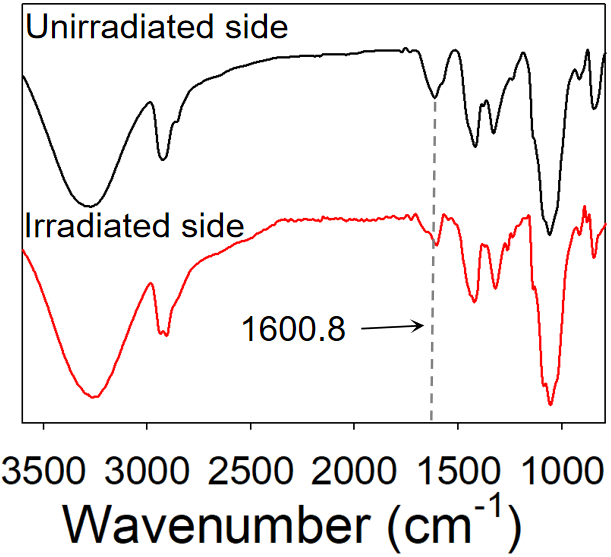


**Figure S1**. FT-IR spectra of the two sides of a MC-3 sample.


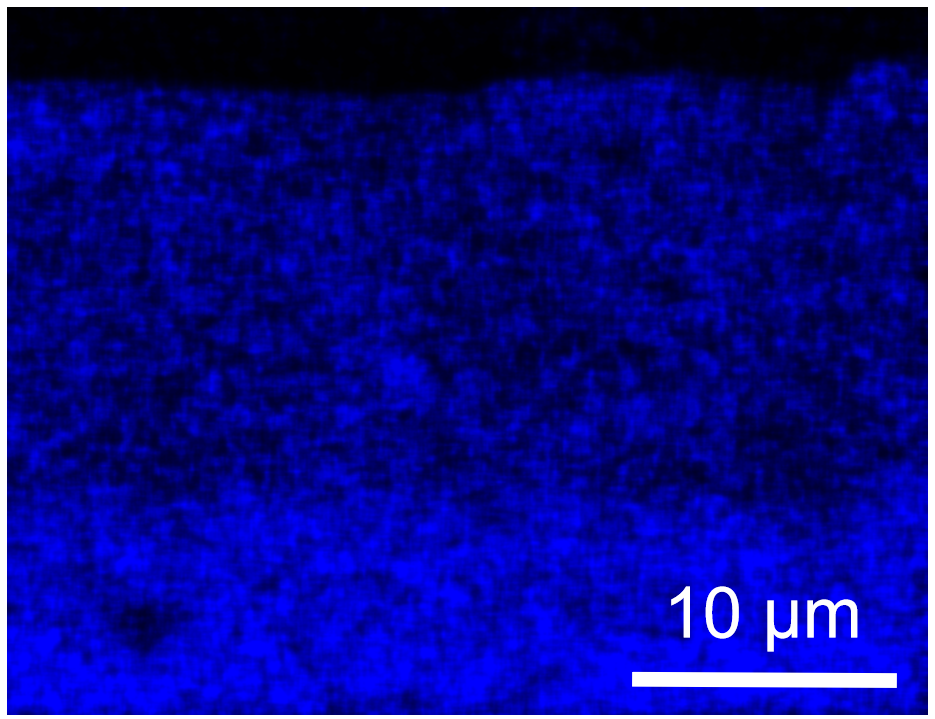


**Figure S2**. XPS of Na element distribution along the thickness direction of a MC-3 sample.


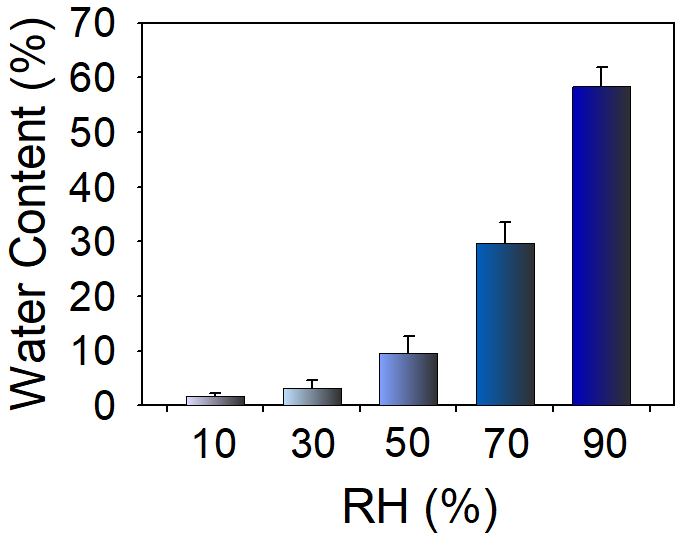


**Figure S3**. Equilibrium water content of MC-3 in environments with different RH.


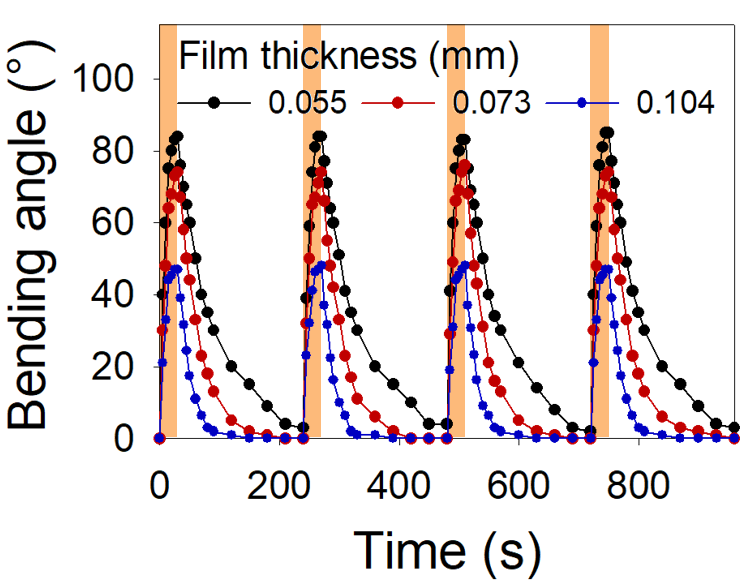


**Figure S4**. Actuating behaviors of MC-3 samples with different thicknesses under cyclic NIR light exposure.


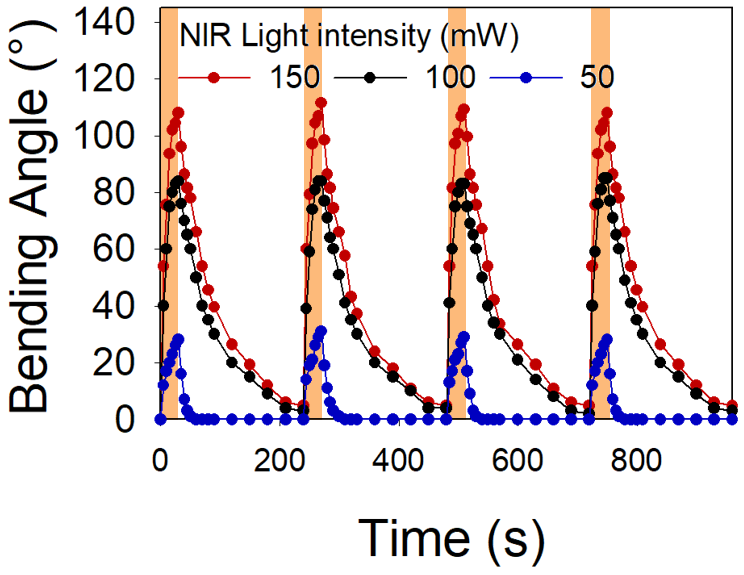


**Figure S5**. Actuating behaviors of MC-3 samples under cyclic NIR light exposure with different intensities.
